# Active and non-active secondary progressive multiple sclerosis patients exhibit similar disability progression: results of an Italian MS registry study (ASPERA)

**DOI:** 10.1007/s00415-024-12621-9

**Published:** 2024-08-27

**Authors:** Clara Grazia Chisari, Maria Pia Amato, Alessia Di Sapio, Matteo Foschi, Pietro Iaffaldano, Matilde Inglese, Salvatore Lo Fermo, Alessandra Lugaresi, Giacomo Lus, Nerina Mascoli, Sara Montepietra, Ilaria Pesci, Rocco Quatrale, Giuseppe Salemi, Valentina Torri Clerici, Rocco Totaro, Paola Valentino, Massimo Filippi, Francesco Patti

**Affiliations:** 1 Department of Medical and Surgical Sciences and Advanced Technologies “GF Ingrassia”, Multiple Sclerosis Center, University of Catania, Catania, Italy; 2 Multiple Sclerosis Unit; Neurology Clinic, Policlinico “G. Rodolico– San Marco”, Catania, Italy; 3 Department of NEUROFARBA, Section of Neurosciences, University of Florence, Florence, Italy; 4 Department of Neurology, Regional Referral Multiple Sclerosis Center, University Hospital San Luigi Gonzaga, Orbassano, Turin, Italy; 5 Department of Neuroscience, Multiple Sclerosis Center, S. Maria delle Croci Hospital of Ravenna, Ravenna, Italy; 6 Department of Biotechnological and Applied Clinical Sciences (DISCAB), University of L’Aquila, L′Aquila, Italy; 7 Department of Translational Biomedicine and Neurosciences (DiBraiN), University of Bari Aldo Moro, Bari, Italy; 8 Department of Neuroscience, Rehabilitation, Ophthalmology, Genetics, Maternal and Child Health (DINOGMI), University of Genoa, Genoa, Italy; 9 IRCCS Ospedale Policlinico San Martino, Genoa, Italy; 10 UOSI Riabilitazione Sclerosi Multipla, IRCCS Istituto Delle Scienze Neurologiche Di Bologna, Bologna, Italy; 11 Department of Biomedical and Neuromotor Sciences, University of Bologna, Bologna, Italy; 12 Multiple Sclerosis Center, Second Division of Neurology, Department of Advanced Medical and Surgical Science, University of Campania Luigi Vanvitelli, Naples, Italy; 13 Neurology Unit, Department of Medicine, S. Anna Hospital, Como, Italy; 14 MS Centre, SMN Hospital, AUSL Reggio Emilia, Reggio Emilia, Italy; 15 Centro Sclerosi Multipla Unità Operativa Neurologia, Azienda Unità Sanitaria Locale, Ospedale Di Vaio, Fidenza, Parma, Italy; 16 Dipartimento Di Scienze Neurologiche, UOC Di Neurologia, Ospedale Dell’Angelo AULSS 3 Serenissima, Venice Mestre, Italy; 17 Department of Biomedicine, Neuroscience and Advanced Diagnostics, University of Palermo, Palermo, Italy; 18 Neuroimmunology and Neuromuscular Diseases Unit, Fondazione IRCCS Istituto Neurologico Carlo Besta, Milan, Italy; 19 Demyelinating Disease Center, San Salvatore Hospital, L′Aquila, Italy; 20 Institute of Neurology, University Magna Graecia, Catanzaro, Italy; 21 Neuroimaging Research Unit, Division of Neuroscience, IRCCS San Raffaele Scientific Institute, Milan, Italy; 22 Neurology Unit, IRCCS San Raffaele Scientific Institute, Milan, Italy; 23 Neurorehabilitation Unit, IRCCS San Raffaele Scientific Institute, Milan, Italy; 24 Vita-Salute San Raffaele University, Milan, Italy; 25 Neurophysiology Service, IRCCS San Raffaele Scientific Institute, Milan, Italy

**Keywords:** Secondary progressive multiple sclerosis, Disease activity, Progression

## Abstract

‘Active’ and ‘non-active’ secondary progressive MS (SPMS) have distinct pathophysiological mechanisms and clinical characteristics, but there is still no consensus regarding the frequency of these MS forms in the real-world setting. We aimed to evaluate the frequency of ‘active’ and ‘non-active’ SPMS in a large cohort of Italian MS patients and the differences in terms of clinical and MRI characteristics and disease progression. This multicenter study collected data about MS patients who have transitioned to the SP form in the period between 1st January 2014 and 31st December 2019 and followed by the MS centers contributing to the Italian MS Registry. Patients were divided into ‘active SPMS’ and ‘non-active SPMS’, based on both reported MRI data and relapse activity in the year before conversion to SPMS. Out of 68,621, 8,316 (12.1%) patients were diagnosed with SPMS. Out of them, 872 (10.5%) were classified into patients with either ‘active’ or ‘non-active’ SPMS. A total of 237 were classified into patients with ‘active SPMS’ (27.2%) and 635 as ‘non-active SPMS’ (72.8%). ‘Non-active SPMS’ patients were older, with a longer disease duration compared to those with ‘active SPMS’. The percentages of patients showing progression independent of relapse activity (PIRA) at 24 months were similar between ‘active’ and ‘non-active’ SPMS patients (67 [27.4%] vs 188 [29.6%]; *p* = 0.60). In the ‘active’ group, 36 (15.2%) patients showed relapse-associated worsening (RAW). Comparison of the survival curves to EDSS 6 and 7 according to disease activity did not show significant differences (*p* = 0.68 and *p* = 0.71). ‘Active’ and ‘non-active’ SPMS patients had a similar risk of achieving disability milestones, suggesting that progression is primarily attributed to PIRA and only to a small extent to disease activity.

## Introduction

Multiple sclerosis (MS) is a chronic demyelinating and neurodegenerative disease of the central nervous system (CNS) with a very complex and poorly predictable disease course [29, 32]. The most common form is the relapsing–remitting (RRMS), in which relapses (defined as new or worsening of previous focal neurologic signs and symptoms) are followed by periods of remission. [8, 42]. In several cases, RRMS typically transit over time toward a secondary progressive (SP) phase, defined by an insidious worsening of neurologic function which could be independent of relapses [42]. RRMS and SPMS have been recently acknowledged as part of a disease continuum each with ‘active’ and ‘not active’ clinical characteristics defined by the presence of relapses with or without magnetic resonance imaging (MRI) activity [8, 10, 26, 28, 29]. The underlying mechanisms driving the transition from RRMS to SPMS have not yet been well established. According to some authors, the RRMS is primarily characterized by an intense inflammatory process, then, during the SPMS phase, neurodegeneration may occur seemingly independent of relapse-associated imaging outcomes (new T2 lesion and/or contrast-enhanced lesions [CELs]) [8]; new evidence demonstrates that 53% of patients with ‘non-active’ disease exhibit positron emission tomography (PET) findings of microglia inflammatory activation [3, 16]. Moreover, evidence suggests that disease activity may be found in patients with SPMS, who can still experience relapses [25, 27].

According to the recent definition, both ‘active’ and’non-active’ SPMS may have distinct pathophysiological mechanisms and clinical characteristics, but there is still no consensus regarding the frequency of these MS forms in real-world setting [17, 18, 25, 26]. Furthermore, as the levels of disease activity and progression are likely to affect therapeutic decisions [19, 33], detailed knowledge of the frequency and characteristics of both ‘active’ and ‘non-active’ SPMS is required to provide suitable treatment strategies. In this respect, a recent re-analysis of two RCTs on the efficacy of interferon-beta and glatiramer acetate versus placebo in progressive MS demonstated that progression may slightly benefit from anti-inflammatory effects [34].

In this view, the aim of this registry-based multicenter study ASPERA (‘Evaluating the clinicAl and MRI characteristics of Secondary Progressive multiplE sclerosis; a registRy-bAsed/multicentric cohort study’) was to evaluate the frequency of ‘active’ and ‘non-active’ SPMS in a large cohort of Italian MS patients and to investigate whether these two groups differ in terms of clinical and MRI characteristics, and, particularly, in disease progression.

## Materials and methods

### Study population

This retrospective multicenter study collected data about MS patients transitioning to the SP form in the period between 1st January 2014 and 31st December 2019 and who are followed by the MS centers contributing to the Italian Multiple Sclerosis and Related Disorders Register.

Patients were attributed to SPMS course according to the retrospective evaluation of neurologists considering a history of gradual progression following an initial relapsing–remitting course [29]. We also defined two groups, ‘active SPMS’ and ‘non-active SPMS’, based on both reported MRI data and relapse activity [29]. According to the presence of disease activity in the year before the conversion to SPMS, patients were classified according to the Lublin criteria (2014) [29] as follows—active SPMS: patients showing at least one relapse or MRI activity defined by at least one CEL and/or new T2 lesions; non-active SPMS: patients showing no clinical relapse and no MRI activity. Differences in clinical, MRI and demographical data after conversion to SPMS were evaluated between ‘active’ and ‘non-active’ SPMS. Moreover, the disease activity in terms of clinical relapse, new or enlarged T2 lesion or CEL at 1 and 2 years after SP conversion was also investigated.

### Inclusion/exclusion criteria

We included in this study all patients with a diagnosis of SPMS in accordance with the Lublin criteria [29], for whom clinical data (relapse and Expaned Disability Status Scale [EDSS]) and MRI data and at least one MRI examination at the time of SP conversion (± 12 months from the date of SP conversion) were available.

### Ethical statement

The Italian MS registry study was approved by the ethical committee at the “Azienda Ospedaliero–Universitaria–Policlinico of Bari” (Study REGISTRO SM001 approved on 08/07/2016) and by the local ethics committees in all participating centers where patients signed a written informed consent that allows to use clinical data for research purposes.

### Definition of outcomes and variables

We calculated the proportion of patients with ‘active’ or ‘non-active’ SPMS who have transitioned to the SP form in the period between 1st January 2014 and 31st December 2019. Clinical activity was defined as the occurrence of at least one relapse during the studied years. Relapses were defined as the occurrence of new neurological symptoms or as the worsening of previous neurological symptoms lasting > 24 h without fever or infection [9, 10] and were diagnosed by the attending neurologist. We defined radiological activity as the appearance of at least one new CEL and/or a “worsening” (new appearance and/or enlargement) on T2-weighted imaging on at least one brain or spinal cord MRI carried out during the observation period with reference to a previously performed scan.

Demographical, clinical (age at MS onset and MS diagnosis, age at SPMS transition, symptoms at onset, EDSS at MS diagnosis and at SPMS transition) and MRI characteristics (number of brain and spine lesions at MS onset and SPMS transition) and treatment choice after conversion to SPMS were compared between ‘active’ and ‘non-active’ SPMS. We also evaluated disease activity (clinical relapse, new or enlarged T2 lesion or CEL) at 12 (T12) and 24 (T24) months after conversion to SPMS.

Progression independent of relapse (PIRA) was evaluated at T24. PIRA was defined as a ≥ 12 week confirmed disability progression; this is referred to as a worsening of 1 point on the EDSS in patients with a baseline EDSS between 3.0 and 5.0 or a 0.5 step in EDSS in patients with a baseline EDSS ≥ 5.5 in the absence of relapse. According to PIRA definition, the relapse-free interval was calculated for a period of at least 12 consecutive months [20]. In addition, relapse-associated worsening (RAW) was calculated according to the presence of a confirmed disability accumulation event (confirmed worsening by 1.0 point or more in EDSS score) which is preceded by any defined relapse in the last 6 months [20].

### Statistical analysis

Statistical analysis was carried out using the statistic package STATA 16.6. Shapiro–Wilk test was used to assess normal distribution. Continuous variables were summarized by the number of observations, mean, standard deviation (SD) and median (range), according to their distribution. Categorical data were presented by absolute and relative frequencies (n and %) or contingency tables. If the assumptions for F or *t* tests were violated, equivalent non-parametric statistics were used. Patients were divided into two groups, ‘active’ and ‘non-active’ SPMS, and compared for demographical, clinical and MRI data. Student’s *t* test or the Mann–Whitney *U* test were applied to analyze differences in continuous measures, while the Fisher’s exact test was used for categorical measure. The association between two quantitative variables were performed through Pearson correlation coefficient or Spearman correlation coefficient, depending on the data distribution.

Repeated measures two-way ANOVA was run to compare clinical and MRI data at different time points. In case of significant effects, pairwise comparisons were carried out applying the Bonferroni correction.

The risks of reaching 24-month confirmed EDSS 6.0 and EDSS 7.0 were evaluated with Kaplan–Meier and Cox regression models, including a binary indicator of group (e.g., ‘active’/’non-active’) as an explanatory variable. Kaplan–Meier estimates were compared across groups through log-rank tests. The variables related with time to EDSS 6.0 and time to EDSS 7.0 on univariable analysis (p ≤ 0.10) were considered for inclusion in multivariable analysis. Cox models were adjusted for the following covariates: sex, age at diagnosis, disease duration, age at SP conversion, EDSS at SP conversion, disease-modifying therapies after SP conversion (yes/no), disease-modifying therapies before conversion to SPMS (yes/no), relapses before conversion to SPMS (yes/no), relaspses after SP conversion (yes/no), MRI activity before conversion to SPMS (yes/no) and MRI activity after conversion to SPMS (yes/no). For all survival models, the proportional hazard assumption was assessed through the scaled Schoenfeld residuals test. The adjusted hazard ratios (HR) and their 95% confidence intervals (CI) were used to interpret the final model. A p value equal to or lower than 0.05 was considered statistically significant.

## Results

Out of 68,621 MS patients recorded in the Italian MS Registry, we identified 8,316 (12.1%) patients who were diagnosed with SPMS. Out of them, in the period between 1st January 2014 and 31st December 2019, 872 (10.5%) could be classified as either ‘active’ or ‘non-active’ SPMS oatients according to the clinical and MRI data, while 7,444 (89.5%) were excluded as considered ‘unclassifiable’.

**Fig. 1:**
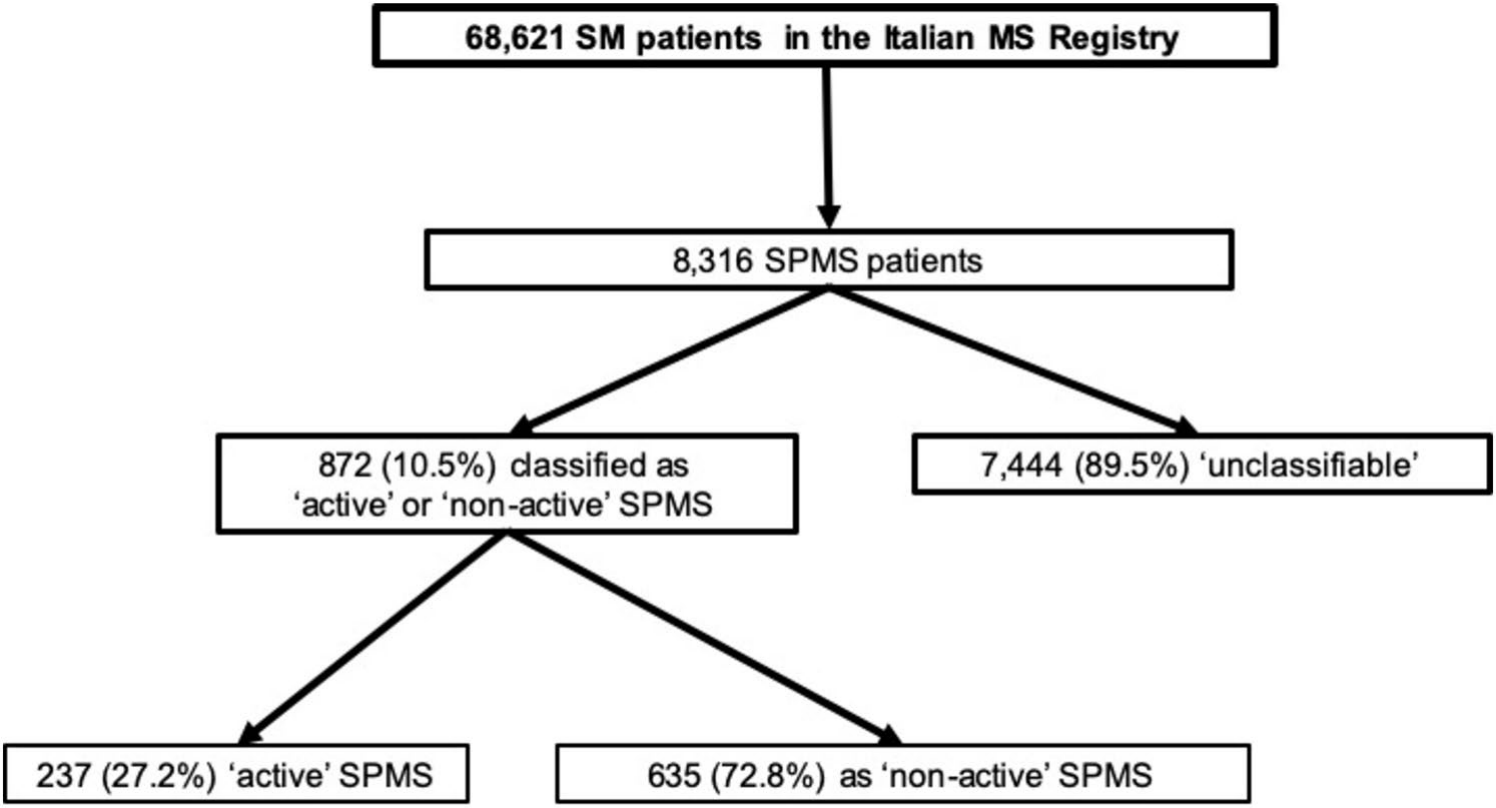
Flowchart of all 8,316 SPMS patients

Out of the 872 SPMS patients with the required data available, 237 (27.2%) were classified as having ‘active SPMS’ and 635 (72.8%) with ‘non-active SPMS’ (Fig. 1).

Particularly, ‘non-active SPMS’ patients were significantly older, with a significant longer disease duration compared to ‘active SPMS’ patients. Patients in the ‘active SPMS’ group had a higher number of Gd-enhanced lesion at MS diagnosis compared to the ‘non-active’ ones (Table 1).

**Table 1:** Demographic and clinical characteristics of the two cohorts

Total 872 *N* (%)	Active SPMS 237 (27.2)	Non-active SPMS 635 (72.8)	*P* value
Female; *N* (%)	155 (65.4)	401 (63.1)	0.8
Age (years); mean ± SD Median (range)	54.2 ± 10.6 46 (41–65)	57.7 ± 14.2 49 (43–70)	*0.01*
Age at onset (years); mean ± SD Median (range)	34.4 ± 10.1 31 (22–46)	33.8 ± 11.9 30 (22–45)	0.5
Disease duration (years); mean ± SD Median (range)	21.5 ± 9.3 12 (10–32)	25.3 ± 13.8 11 (10–38)	*0.001*
EDSS at diagnosis of MS; mean ± SD Median (range)	2.5 ± 1.2 1.5 (0.0–3.5)	2.7 ± 1.6 1.5 (0.0–3.5)	*0.08*
N. of relapses at diagnosis of MS; mean ± SD Median (range)	1.6 ± 1.5 1 (1–3)	1.5 ± 1.3 1 (1–3)	0.3
N. of CELs at diagnosis of MS; mean ± SD Median (range)	± 0.7 0 (0–4)	0.5 ± 0.7 0 (0–3)	*0.001*
N. of new or enlarged T2-weighted lesions at diagnosis of MS; mean ± SD Median (range)	0.4 ± 0.1 0 (0–2)	0.5 ± 0.3 0 (0–2)	0.2

*EDSS* expanded disability status scale, *CELs* contrast-enhanced lesions, *SD* standard deviation, *SPMS* secondary progressive multiple sclerosis

The percentages of patients showing PIRA at 24 months were similar between ‘active’ and ‘non-active’ SPMS patients (67 [27.4%] in ‘active’ compared to 188 [29.6%] in ‘non-active’ SPMS; *p* = 0.6). Morover, in the ‘active’ group, a significant higher percentage of patients (36 [15.2%] vs 8 [1.4%], *p* < 0.001) showed RAW compared to the ‘non-active group’.

At T12 and T24, ‘active SPMS’ showed a reduction in terms of the proportions of patients reporting relapses (79.3% vs 26.6% vs 17.7%, *p* < 0.001) and presenting with MRI activity (33.3% vs 13.5% vs 19.4%, *p* < 0.01) compared to the time of SPMS conversion.

We also found that, in the ‘non-active’ group, 23 (3.6%) showed disease activity in terms of relapses and/or MRI activity at 24 months. No differences were observed in terms of relapses and MRI activity in the ‘non-active SPMS’ group at T12 and T24 time points (Table 2).

**Table 2:** Differences in terms of confirmed disability worsening in ‘active and ‘non-active’ SPSM patients

Total 872 *N* (%)	Active SPMS 237 (27.2)				Non-active SPMS 635 (72.8)			
	At SPMS conversion (A)	T12 (B)	T24 (C)	ANOVA *p* value^*^	At SPMS conversion (A)	T12 (B)	T24 (C)	ANOVA *p* value^*^
EDSS mean ± SD Median (range)	5.3 ± 2.5 4.5 (3.5–7)	5.1 ± 2.8 4.5 (3.5–7)	5.7 ± 2.2 5 (3.5–7.5)	0.3	5.5 ± 3.2 4.5 (4–7)	5.6 ± 3.5 5 (4–7.5)	5.8 ± 3.1 5 (4.5–7.5)	0.3
No. of relapses; mean ± SD Median (range)	1.4 ± 0.4 1 (0–4)	1.0 ± 0.7 1 (0–3)	0.9 ± 0.6 0 (0–2)	A vs C: 0.06	0	0.4 ± 0.1 0 (0–2)	0.5 ± 0.2 0 (0–3)	0.7
No. of patients reporting clinical relapses; *N* (%)	188 (79.3)	63 (26.6)	42 (17.7)	A vs B vs C: < 0.001	0	3 (0.5)	5 (0.8)	0.4
No. of CELs; mean ± SD Median (range)	0.6 ± 0.4 0 (0–2)	0.4 ± 0.1 0 (0–1)	0.3 ± 0.1 0 (0–1)	0.2	0	0.2 ± 0.1 0 (0–1)	0.3 ± 0.2 0 (0–1)	0.8
No. of new or enlarged T2-weighted lesions; mean ± SD Median (range)	0.8 ± 0.3 0 (0–2)	0.6 ± 0.2 0 (0–2)	0.5 ± 0.3 0 (0–1)	A vs C: 0.07	0	0.5 ± 0.4 0 (0–2)	0.3 ± 0.1 0 (0–1)	0.3
No. of patients with MRI activity; *N* (%)	79 (33.3)	32 (13.5)	46 (19.4)	A vs B vs C: < 0.01	0	12 (1)	5 (0.8)	0.5

*EDSS* expanded disability status scale, *CELs* contrast-enhanced lesions, *SD* standard deviation, *SPMS* secondary progressive multiple sclerosis

*After Bonferroni correction

**Fig. 2:**
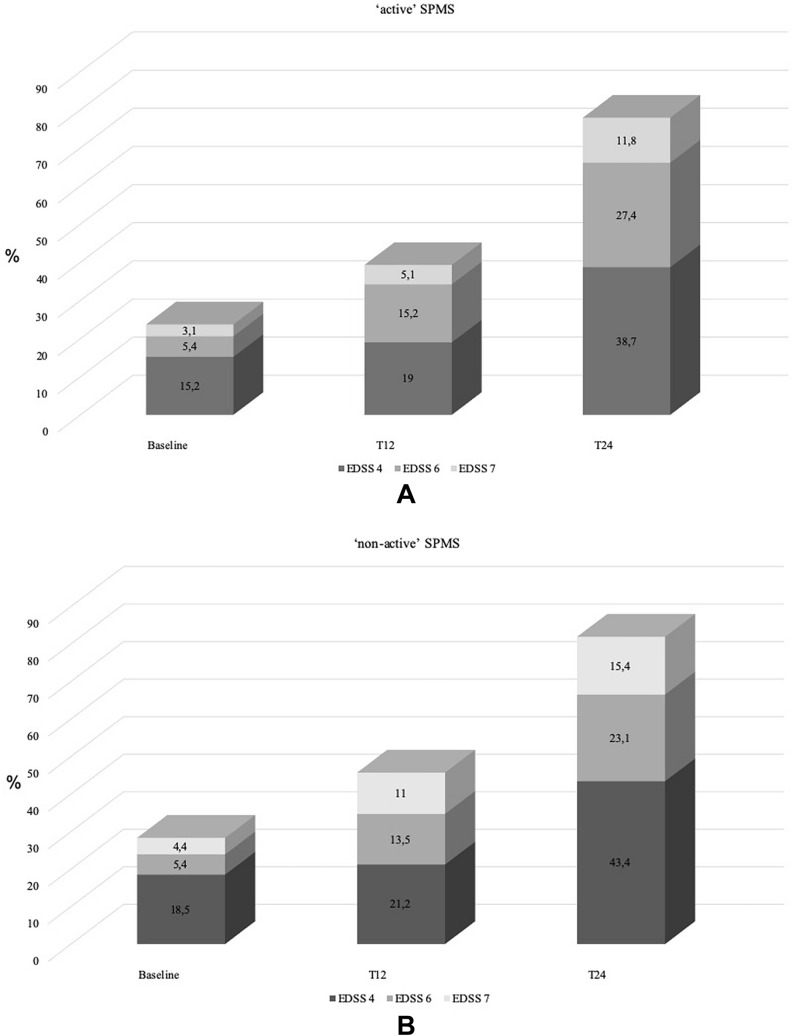
Distribution of patients with EDSS 4, 6 and 7 at the time con SPMS conversion (baseline), after 12 (T12) and 24 (T24) months in ‘active’, **A** and non-active’, **B** SPMS EDSS: Expanded Disability Status Scale; SPMS: secondary progressive multiple sclerosis

Moreover, at T12 and T24, both ‘non-active’ and ‘active’ SPMS groups showed a significant increase in the proportions of patients reaching EDSS 6.0 (5.4% vs 13.5% vs 23.1%, *p* = 0.05 in ‘non-active’; 5.4% vs 15.2% vs 27.4%, *p* = 0.03 in ‘active’ SPMS) and 7.0 (4.4% vs 11% vs 15,4%, *p* = 0.06 in ‘non-active’; 3.1% vs 5.1% vs 11.8%, *p* = 0.07) at SPMS conversion (Fig. 2).

**Fig. 3:**
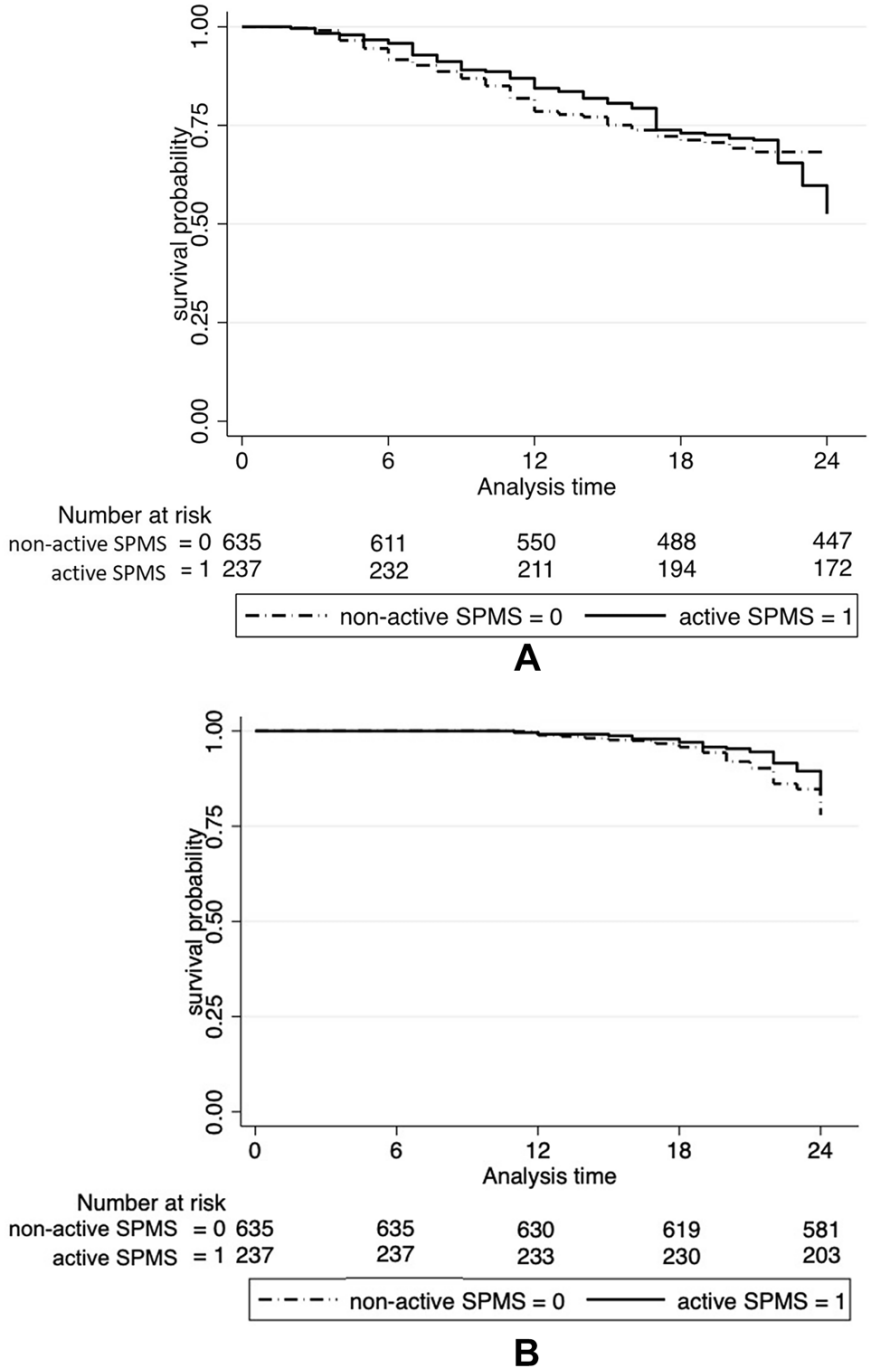
Kaplan–Meier curves for the time of reaching EDSS 6 (**A**) and EDSS 7 (**B**) in the ‘active’ and non-active’ SPMS EDSS: Expanded Disability Status Scale; SPMS: secondary progressive multiple sclerosis

In the ‘active’ SPMS group, the median times to EDSS 6.0 and 7.0 (89 [IQR 54–108] and 101 [IQR 75–142] months, respectively) were statistically similar to those observed in the ‘non-active’ SPMS group (79 [IQR 51–101] and 95 [IQR 62–123] months, respectively; *p* = 0.70). Comparison of the survival curves to EDSS 6.0 and 7.0 according to disease activity did not show significant differences (*p* = 0.68 and *p* = 0.71) (Fig. 3 A-B). There is no evidence of an association between the clinical adjustment variables and the time of reaching the EDSS 6.0 and 7.0.

## Discussion

This multicenter retrospective Italian Registry study estimated the frequency of ‘active’ and ‘non-active’ SPMS and attempted to identify demographical and clinical differences between the two subgroups, based on real-world data.

One of the main findings of this study was that the percentages of patients showing PIRA were similar in ‘active’ and ‘non-active’ SPMS. Moreover, the comparison of the survival curves to EDSS 6.0 and 7.0 according to disease activity did not show significant differences. This may reflect the main contribution of clinical PIRA on disease progression, with a small amount originating from worsening related to relapses, as previously reported [43]. Accordingly, in the whole cohort, the percentage of patients showed RAW was overall low, although significantly higher in the ‘active’ group. To confirm this, data from randomized clinical trials indicated that PIRA events constituted roughly 70–90% of all disability accrual events over a follow-up period of 2–10 years [9, 20, 43]. New emerging evidence suggested that the progressive accumulation of disability may be partly independent of clinical relapse and focal radiological activity from early in the disease course [39]. In fact, more recent studies have put the spotlight on the role of chronic active lesions as marker of a more diffuse smoldering pathological process affecting the entire CNS [15]. Indeed, PET studies have demonstrated the presence of active inflammatory cells at chronic active lesion borders, indicating an association between smoldering inflammation and disability also in patients who did not show CELs [6, 38]. Thus, the distinction between ‘active’ and ‘non-active’ SPMS could be outdated and replaced with a new concept of MS that progresses along a continuum from relapsing to progressive forms, as proposed by several authors [24, 40].

Recently, the definition of “therapeutic lag” has been also introduced, referring to the time required for the current anti-inflammatory drugs to exert their effect on the degenerative process [34]. Particularly, according to a recent study, in progressive MS the "therapeutic lag " for low-efficacy treatments in progression can take 2–2.5 years, while clinial trials typically last only 2 years. For this reason, the authors concluded that PIRA may not be fully independent of the factors influencing relapse activity [34]. In line with this, our data show that non-active patients do not remain so forever, as part of our patients were active at T12 and T24, although in a smaller proportion than previously active MS. Therefore activity is not fixed, but varies with time, and the residual presence of activity correlates with effectiveness of treatment. Although this new approach seems to clash with the evidence that the current DMTs are more efficacious on ‘active’ than ‘non-active’ SPMS [4, 37, 41, 43], it has recently introduced a new perspective on MS progression, viewing it as a spectrum shaped by the balance between overlapping pathological and reparative/compensatory processes [24].

Interestingly, according to the definition from Lublin et al. 2014 [29], in our study in the 10.5% of 8,316 SPMS patients who could be classified as ‘active’ or ‘non-active’, only less than one-third could be categorized as ‘active’. A recent analysis of clinical and demographical data of 61,900 MS patients obtained from MS registries in the Czech Republic, Denmark, Germany, Sweden and the United Kingdom (UK) showed that many MS patients still clinically defined as RRMS are more likely to have clinical features compatible with an SPMS course, thus suggesting a systematic underestimation of active SPMS in registry-based MS populations [12]. Similarly, a real-world study from the German MS register (GMSR) recorded low frequencies of ‘active’ and non-active’ SPMS [13]. Notably, the conversion from the relapsing–remitting to the progressive form of MS lacks clear delineation as the transition occurs seamlessly without defined time points [11, 14, 23]. Indeed, because of the observed progressive brain loss in RRMS and relapses persisting into the SPMS [1], some authors suggested that a more accurate portrayal of MS involves a continuum rather than distinct stages [15, 36]. However, the prevalent idea that relapses and progression necessitate distinct treatment strategies have influenced all levels of MS drug development including the current labeling of DMTs and have also supported the relevance of a “two-staged”model of MS.

We also demonstrated that 21.6% of 872 SPMS patients experienced relapses and less than 10% showed MRI activity in the year prior to the SP conversion. Accordingly, similar percentages of disease activity in cohorts of SPMS patients were found in two French studies, one of them from a population-based registry [2, 31]. It is known that regular MRI evaluations have an important role in the classification of disease activity, particularly in the SPMS form [21], since progressive course typically has low relapse rate [35]. Indeed, a recent position paper highlighted the importance of assessing the extent of the ongoing inflammatory component of MS with regular MRI evaluations, particularly when making treatment decisions [36].

Furthermore, in our study, at T12 and T24, the ‘active’ MS group showed a slight reduction of the number of the relapses and of new or enlarged T2 lesions. This finding is in line with several studies showing a reduction of the relapse rate with increasing age [5, 7]. Therefore, aging is one of the main factors contributing to non-relapse disease progression [24, 30]. Evidence of this is that the microglia activation in periplaque white matter was found to be strongly modulated by aging, with higher microglia numbers in younger than in older MS patients [22].

On the contrary, both ‘active’ and ‘non-active’ SPMS in our cohort showed a worsening of progression, expressed as percentages of patients achieving the disability milestones, EDSS 6 and 7. This was not surprising considering that, at the time of data extraction, no specific drugs were available for the treatment of secondary progressive forms.

The main limitation was that the retrospective design of the current study may reduce the statistical power of our results. As a consequence, we did not apply uniform criteria for diagnosing SPMS and the detection of disease activity among the participating centers and, thus, the assignment to the SP course to the ‘active’ or ‘non-active’ group was based on the patients’ history, disability status and the clinicians’experience. Second, MRI protocols were not standardized among all the centers participating to the study, resulting in a potential lack of homogeneity in the imaging evaluations. However, the MRI schedule is quite similar for all centers, being essentially based on standard clinical practice. Nevertheless, this study explored a wide and well-defined study population and the real-world design may have limited the selection bias and ensured the generalizability of the results. Moreover, we indicated that the classification rate in the registry is low, with only 10.5% of 8,316 patients categorized as ‘active’ or ‘non-active’. This was primarily due to the insufficient MRI data included in the registry. In addition, the limited treatment options available for SPMS at the time of data extraction might have led neurologists to delay the assignment of patients to an SPMS disease course, insofar as this may make them ineligible for most available DMTs. Finally, the conversion to SPMS typically occurs without specific time points and, therefore, the actual date of transition to the progressive form cannot be exactly established.

In conclusion, this study offers insights into the frequency and the clinical features of SPMS patients based on disease activity in a real-world setting and it is the first study tp compare ‘active and ‘non-active’ SPMS in terms of disease progression. According to our data, both ‘active’ and ‘non-active’ SPMS patients had similar risk of achieving disability milestones, suggesting that progression is primarily attributed to neurodegeneration, as expressed by PIRA, and only to a small extent to disease activity, in terms of clinical relapses and/or MRI activity. Thus, we could hypothesize that further factors could accomplish more diffuse inflammation, which, on the basis of PIRA and EDSS deterioration, we attributed to neurodegenerative processes. Finally, future studies may attempt to evaluate the impact of the new approved drugs for SPMS on disability accrual, also taking into account the underlying inflammatory events, in terms of relapses and MRI activity.

## Data Availability

The dataset is available on reasonable request to the corresponding author.
